# Postoperative Insulin-Like Growth Factor 1 Levels Reflect the Graft’s Function and Predict Survival after Liver Transplantation

**DOI:** 10.1371/journal.pone.0133153

**Published:** 2015-07-17

**Authors:** Daniele Nicolini, Federico Mocchegiani, Gioia Palmonella, Martina Coletta, Marina Brugia, Roberto Montalti, Giammarco Fava, Augusto Taccaliti, Andrea Risaliti, Marco Vivarelli

**Affiliations:** 1 Division of Hepatobiliary and Transplant Surgery, Department of Gastroenterology and Transplantation, Polytechnic University of Marche, Ancona, Italy; 2 Division of Endocrinology, Department of Clinical and Molecular Sciences, Polytechnic University of Marche, Ancona, Italy; 3 Division of Laboratory Medicine, Department of Services, A.O.U. “Ospedali Riuniti”, Ancona, Italy; 4 Division of Gastroenterology, Department of Gastroenterology and Transplantation, A.O.U. “Ospedali Riuniti”, Ancona, Italy; 5 Division of Liver and Kidney Transplant Surgery, Department of Medical and Biological Sciences, University of Udine, Udine, Italy; Texas A&M Health Science Center, UNITED STATES

## Abstract

**Background:**

The reduction of insulin-like growth factor 1 (IGF-1) plasma levels is associated with the degree of liver dysfunction and mortality in cirrhotic patients. However, little research is available on the recovery of the IGF-1 level and its prognostic role after liver transplantation (LT).

**Methods:**

From April 2010 to May 2011, 31 patients were prospectively enrolled (25/6 M/F; mean age±SEM: 55.2±1.4 years), and IGF-1 serum levels were assessed preoperatively and at 15, 30, 90, 180 and 365 days after transplantation. The influence of the donor and recipient characteristics (age, use of extended criteria donor grafts, D-MELD and incidence of early allograft dysfunction) on hormonal concentration was analyzed. The prognostic role of IGF-1 level on patient survival and its correlation with routine liver function tests were also investigated.

**Results:**

All patients showed low preoperative IGF-1 levels (mean±SEM: 29.5±2.1), and on postoperative day 15, a significant increase in the IGF-1 plasma level was observed (102.7±11.7 ng/ml; p<0.0001). During the first year after LT, the IGF-1 concentration remained significantly lower in recipients transplanted with older donors (>65 years) or extended criteria donor grafts. An inverse correlation between IGF-1 and bilirubin serum levels at day 15 (r = -0.3924, p = 0.0320) and 30 (r = -0.3894, p = 0.0368) was found. After multivariate analysis, early (within 15 days) IGF-1 normalization [Exp(b) = 3.913; p = 0.0484] was the only prognostic factor associated with an increased 3-year survival rate.

**Conclusion:**

IGF-1 postoperative levels are correlated with the graft’s quality and reflect liver function. Early IGF-1 recovery is associated with a higher 3-year survival rate after LT.

## Introduction

The liver plays a pivotal role in the homeostasis of the growth hormone (GH)/insulin growth factor-1 (IGF-1) axis and also secretes more than 90% of circulating IGF-1 and mediates the effects of GH on cellular cycle regulation and metabolism[1, 2]. It is known that serum IGF-1 concentration is dependent on several factors, such as general endocrine balance and nutritional status, as exhibited by the wide range of normal values and the age-related decline in healthy individuals[3, 4]. In the case of liver cirrhosis, the impaired synthetic capacity of the hepatocellular mass, combined with the reduction of GH liver receptors lead to a decrease in IGF-1 and insulin growth factor 1 binding proteins (IGFBPs) serum levels[5–7]. Because of the lack of a negative feedback blockade from circulating IGF-1, a simultaneous increased secretion of GH is also observed[8]. Several authors demonstrated that the degree of hormonal imbalance is directly related to the severity of liver dysfunction, as expressed by the Model for End Stage Liver Disease (MELD) and Child-Pugh scores[9–11], and have identified IGF-1 as a reliable prognostic tool in patients with chronic liver disease[11, 12].

Studies on pediatric and adult patients have demonstrated a dramatic recovery of the GH/IGF-1 axis after liver transplantation (LT), which suggests that IGF-1 serum levels can be useful in monitoring the graft’s function in the postoperative period[13–16]. Bassanello and colleagues observed an increase in IGF-1 serum levels starting 30 minutes after reperfusion of the graft and a normalization of hormonal values between one week and 1 month after surgery, accompanied with a complete hepatic recovery of the 15 recipients included in the study[17].

Immediate and long-term function of the liver graft after transplantation is directly correlated with the quality of the donor liver, as well as multiple host-related variables that may affect the intraoperative course and the initial posttransplant recovery period[18]. Serial measurements of routine liver function tests, such as liver specific transaminases and prothromin activity (PT), are useful in discriminating an initial graft’s poor function, but they may be inadequate for identifying more subtle changes in liver synthetic capability, resulting in a failure to predict the long-term outcome of the transplant.

The aim of this study is to test the reliability of IGF-1 in estimating the graft’s functional reserve during the first year after LT. The kinetics of recovery of normal IGF-1 plasma levels was also investigated to identify a new prognostic tool for predicting the medium-term outcome of LT.

## Materials and Methods

From April 2010 to May 2011, 44 adult liver transplant candidates were prospectively considered for this study. Subjects with human immunodeficiency virus (HIV) infection (8 patients), those with acute liver failure (ALF, 3 patients), and those who were listed for re-LT (1 patient) or were affected by any endocrine disorders not related to liver disease (1 patient) were excluded. Thirty-one cirrhotic patients were enrolled and followed for 3 years after surgery. In Table 1, the demographic and clinical characteristics of the study population in terms of age, sex, primary diagnosis for LT, presence of hepatocellular carcinoma (HCC), waiting list time before LT, Child-Pugh and Model for End Stage Liver Disease (MELD) scores are reported. Every transplant procedure was performed preserving the retrohepatic vena cava according to the piggyback technique, and immunosuppression was initially based on a once daily dose of Tacrolimus associated with Everolimus starting on postoperative day 14. Steroid therapy was discontinued 3 months after transplantation. The graft’s quality was defined according to the cumulative number of risk factors proposed by Gruttadauria et al.[19]. In particular, an extended criteria donor (ECD) was defined when 2 or more of the following risk factors were present: (1) age > 60 years; (2) macrovesicular steatosis > 30%; (3) prolonged intensive care unit stay (> 7 days); (4) hemodynamic risk factors, including prolonged hypotension (systolic blood pressure >60 mmHg for more than 2 hours), use of dopamine >10 μg/kg/minute for more than 6 hours to sustain blood pressure, and need for 2 inotropic drugs to sustain donor blood pressure for more than 6 hours; (5) cold ischemic time >12 hours; and (6) hypernatremia (Na peak >160 mEq/L) before aortic cross clamp. The donor risk index[20] (DRI) and the donor age x recipient MELD score (DMELD)[21] were also calculated. Peripheral venous blood samples were collected 4–6 hours prior to transplantation and on postoperative days (POD) 15, 30, 90, 180 and 365. The assays were performed within 24 h from blood collection. GH and IGF-1 serum levels were measured by a solid-phase, enzyme-labeled chemiluminescent immunometric assay (Immulite 2000, Siemens). IGF-1 kit was calibrated against the World Health Organization International Reference Reagent for IGF-I (87/518), and reference ranges were established according to the study performed by Elmlinger and colleagues[22]. Human GH kit was calibrated against the World Health Organization International Reference Reagent for GH (98/574), and reference intervals were up to 3 ng/mL for males and up to 8 ng/mL for females according to the Immunolite 2000 user manual[23]. Other routine liver function tests (LFTs), including aspartate aminotransferase (AST), alanine aminotransferase (ALT), serum albumin, total bilirubin, gamma-glutamyl transferase (gGT), alkaline phosphatase (ALP) and prothrombin time (PT), were collected every day during the patient’s hospital stay and then at every scheduled visit. A correlation analysis between postoperative IGF-1 levels and routine LFTs was carried out at each of the time points considered. The following clinically relevant postoperative complications were recorded: time and cause of death, acute cellular rejection (ACR), primary nonfunction (PNF), early allograft dysfunction[24] (EAD) and biliary complication (BC). The influence of every clinical or donor-related variable on postoperative hormonal plasma levels was analyzed. The prognostic role of the early (within 15 days) recovery of IGF-1 normal levels and the impact of the following variables on patient survival were also tested by univariate analysis: recipient age >60 years, donor age >65 years, ECD score ≥2, detectable HCV-RNA at transplantation, presence of HCC, total ischemia time longer than 10 hours, occurrence of EAD, DRI >1.7 and DMELD>1300. The Ethical Committee of "Ospedali Riuniti" Hospital approved this study. The Ethical Committe stated that written consent was not needed because there was no intervention proposed by the researchers, and blood samples were taken at the time prescribed by routine clinical practice. None of the transplant donors was from a vulnerable population, and all donors or next of kin provided written informed consent that was freely provided.

**Table 1:** Baseline recipients’ and donors’ characteristics. Continuous values are expressed as the mean ± standard error of the mean. LT, liver transplantation; MELD, Model for End-Stage Liver Disease; PBC, primary biliary cirrhosis; ECD, extended criteria donor; DMELD, donor age x recipient MELD.

Variable	All patients (n° 31)
Recipient Age at LT (years)	55.2 ± 1.4
Recipient Male Gender (n°;%)	25 (80.6)
Biochemical MELD Score	15.3 ± 0.9
Child-Pugh Class	
A (n°;%)	5 (16.1)
B (n°;%)	16 (51.6)
C (n°;%)	10 (32.3)
Etiology of Cirrhosis	
HCV (n°;%)	14 (45.2)
HBV (n°;%)	6 (19.3)
Alcoholic (n°;%)	4 (12.9)
Cryptogenic (n°;%)	4 (12.9)
PBC (n°;%)	3 (9.7)
Presence of HCC (n°;%)	11 (35.5)
Donor Age (years)	67.2 ± 2.1
ECD-score ≥ 2 (n°;%)	16 (51.6)
Donor Risk Index	1.9 ± 0.04
Cold Ischemia Time (hours)	7.7±0.4
Warm Ischemia Time (minutes)	32.8 ± 0.02
DMELD	1029.1 ± 78.3

### Statistics

Continuous variables were reported as the mean and standard error of the mean (SEM) or median and range as appropriate. Categorical variables were reported as numbers and percentages and were compared with the Fisher’s exact test. Differences between continuous variables were compared with Student’s t test. The statistical analysis of the GH/IGF-1 axis changes over time was performed using Student’s t test for paired samples. The Pearson’s *r* correlation test was employed to assess the association between IGF-1 levels and LFTs. Patient survival was calculated from the day of surgery to death or to the last follow-up visit. The impact of each variable on patient survival was analyzed by Kaplan Meier methods, and subgroups were compared with the Log-rank test. The risk factors affecting survival with a P value equal to or less than 0.2 after univariate analysis were included in a multivariate Cox proportional hazard regression model to identify the independent prognostic factors. A two-sided P value equal to or less than 0.05 was considered statistically significant in all cases. Statistical analysis was performed using MedCalc software for Windows (version 12.5.0).

## Results

### Hormonal trend during the first year after LT and correlation with LFTs

**Fig 1:**
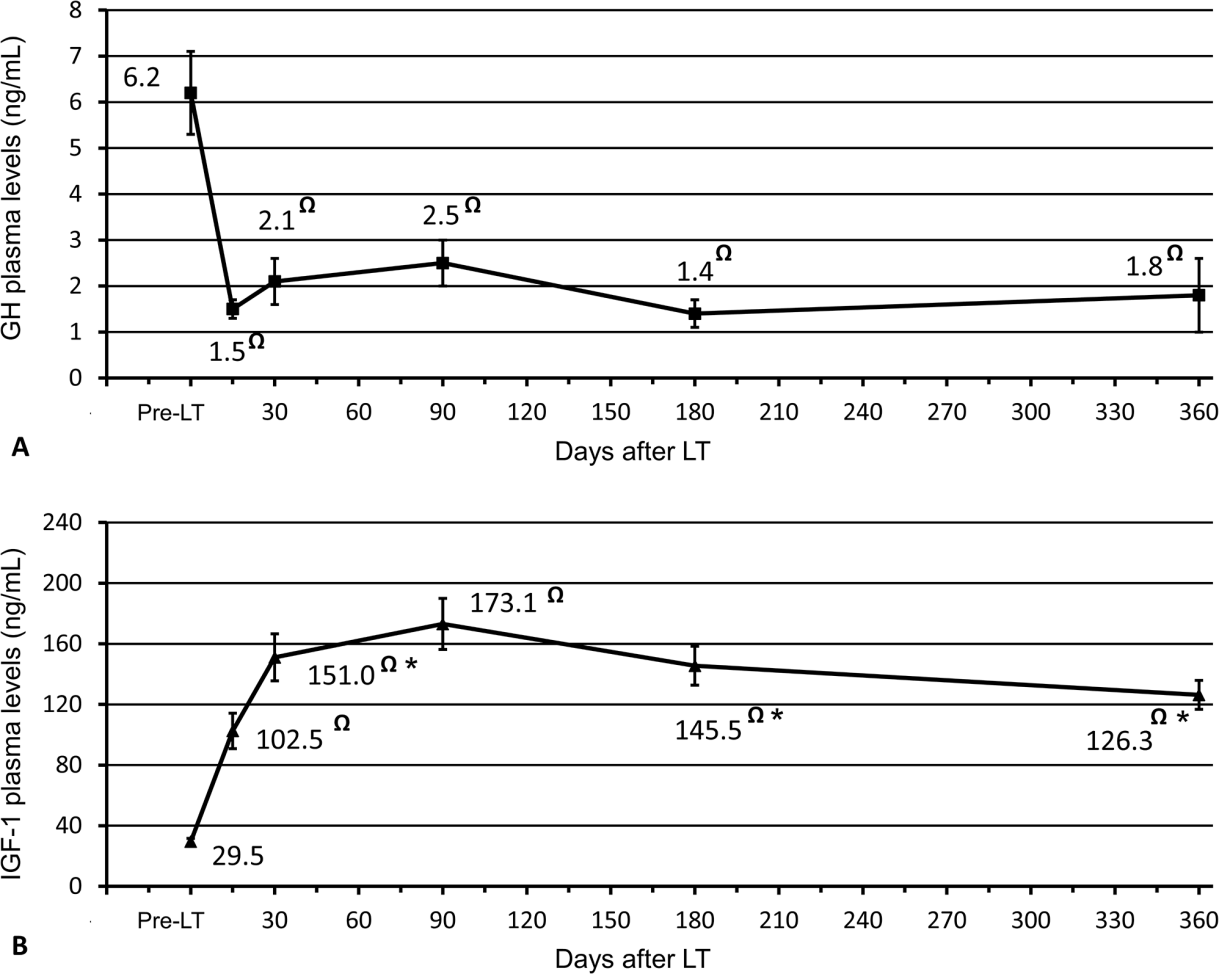
(A) Serum levels of growth hormone (GH) and (B) insulin-like growth factor 1 (IGF-1) before and after liver transplantation. Values are expressed as the mean ± standard error of the mean. Student’s t test for paired samples was employed to investigate significant differences in hormonal values over time. * p <0.05 compared with the previous value; ^**Ω**^ p <0.01 compared with the pre-LT value. **Abbreviations:** IGF-1, insulin-like growth factor 1; LT, liver transplantation; GH, growth hormone.

Nineteen (61.3%) out of 31 patients had high preoperative GH plasma levels (mean±SEM: 6.3±0.9 ng/mL). On postoperative day 15, GH levels showed an abrupt reduction (mean±SEM: 1.5±0.2 ng/mL) and remained substantially stable afterward (Fig 1A). One year after LT, 3 (12.5%) out of 24 survivor patients had pathological GH levels. All patients showed low preoperative IGF-1 levels (mean±SEM: 29.5±2.1 ng/mL). The etiology of liver cirrhosis did not influence basal IGF-1 levels, but a significant inverse correlation between the degree of liver dysfunction assessed by the MELD score and IGF-1 values before LT was observed (r coefficient = -0.3616; p = 0.0456). On day 15, a significant increase in IGF-1 plasma levels was noticed (102.7±11.7 ng/mL; p<0.0001), and 18 out of 31 recipients corrected the IGF-1 levels to normal values. IGF-1 levels continued to rise, reaching a peak 3 months after LT (173.1±16.9 ng/mL), but from then on, a decrease in mean hormonal values was observed (Fig 1B). By the end of 1 year, the mean serum IGF-1 levels was 126.3±9.6 ng/mL, and 21 of the 24 surviving patients maintained normal IGF-1 levels according to reference hormonal ranges. No significant correlation was observed between GH and IGF-1 serum levels at any of the considered time points. The results of the correlation analysis between postoperative IGF-1 levels and main LFTs are shown in Table 2. Within the first month after LT, a significant inverse correlation between IGF-1 and bilirubin serum levels on postoperative day 15 (r = -0.392, p = 0.0320) and 30 (r = -0.389, p = 0.0368) was observed. Moreover, a positive correlation was noticed between IGF-1 and serum albumin on POD 30 (r = 0.595 p = 0.001) and 90 (r = 0.555; p = 0.003). No other statistically significant correlation was found for AST, gGT, ALP and PT at any of the considered time points.

**Table 2:** Correlation analysis between main liver function tests and IGF-1 plasma levels at different time points. IGF-1, insulin-like growth factor 1; POD, postoperative day; C.I. confidence interval.

Liver Function Test	Preoperative		POD 15		POD 30		POD 90		POD 180		POD 365	
	*r* value (95% C.I.)	*p value*	r value (95% C.I.)	*p value*	r value (95% C.I.)	*p value*	r value (95% C.I.)	*p value*	r value (95% C.I.)	*p value*	r value (95% C.I.)	*p value*
Prothrombin time (%)	-0.260 (-0.562–0.104)	0.158	0.316 (-0.049–0.607)	0.088	0.185 (-0.195–0.516)	0.338	0.352 (-0.032–0.646)	0.072	0.323 (-0.131–0.645)	0.164	0.323 (-0.103–0.649)	0.133
Total Bilirubin (mg/dL)	-0.071 (-0.415–0.290)	0.703	-0.392 (-0.659–0.037)	0.032	-0.389 (-0.661–0.027)	0.037	-0.166 (-0.508–0.220)	0.398	0.110 (-0.363–0.537)	0.655	-0.097 (-0.490–0.328)	0.660
Serum Albumin (g/dL)	-0.172 (-0.496–0.194)	0.354	0.230 (-0.142–0.545)	0.230	0.595 (0.293–0.790)	0.001	0.555 (0.213–0.775)	0.003	0.235 (-0.207–0.597)	0.292	0.218 (-0.214–0.578)	0.318
Aspartate aminotransferase (U/L)	0.240 (0.124–0.548)	0.192	-0.245 (-0.556–0.127)	0.192	0.128 (-0.250–0.472)	0.508	-0.281 (0.550–0.180)	0.281	0.354 (-0.079–0.675)	0.106	-0.223 (-0.589–0.219)	0.318
γ-glutamyl transpeptidase (U/L)	-0.080 (-0.423–0.282)	0.668	-0.077 (-0.426–0.291)	0.684	-0.245 (-0.561–0.133)	0.199	-0.097 (-0.460–0.294)	0.631	-0.163 (-0.547–0.277)	0.468	-0.023 (-0.440–0.402)	0.919

### Correlation between donor’s characteristics and IGF-1 postoperative values

**Fig 2:**
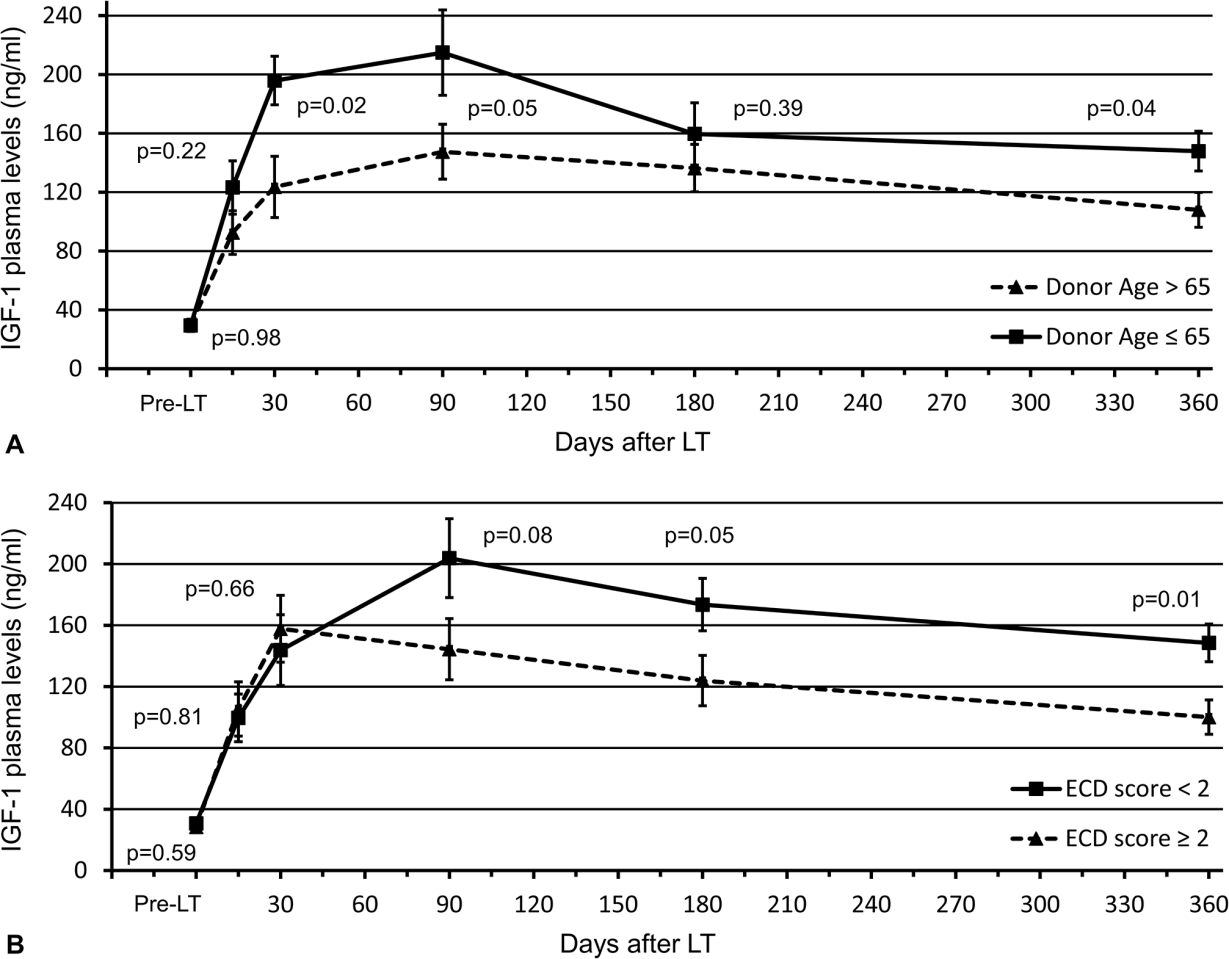
Differences in insulin-like growth factor 1 (IGF-1) serum levels after liver transplantation according to (A) donor age and (B) extended criteria donor (ECD) score. Values are expressed as the mean ± standard error of the mean. Student’s t test for unpaired samples was employed to investigate significant differences between hormonal values at different time points. **Abbreviations:** IGF-1, insulin-like growth factor 1; LT, liver transplantation; ECD, extended criteria donor.

The mean donor age was 67.2±2.1 years, and 20 (64.5%) out of 31 liver grafts were procured from donors aged greater than 65 years. Starting from the first month after LT, IGF-1 serum levels remained significantly lower in recipients transplanted with older donors (>65 years), and this difference was maintained for up to 1 year after transplantation (Fig 2A). According to the ECD score, 4 (12.9%) patients received a liver from a donor considered to be ideal, 11 (35.5%) patients were transplanted with liver grafts with 1 risk factor, and the remaining 16 (51.6%) donors had 2 or more criteria of marginality. As presented in Fig 2B, IGF-1 plasma concentration was noticeably impaired in patients who received a donor with an ECD score ≥2 starting from POD 90. Twenty-five (80.6%) out of 31 donors had a DRI equal to or greater than 1.7 (mean±SEM: 1.9±0.04), and the mean DMELD value was 1029.1±78.3. No correlation between these prognostic models and postoperative IGF-1 levels was found.

### Clinical outcome and IGF-1 plasma levels

The minimum follow-up of each patient included in the present study was 3 years, and none of the patients died within 15 days of transplantation or was retransplanted during the follow-up period. Twenty-nine (93.5%) out of 31 patients were discharged after a median time of 16 days after surgery (range: 8–70 days), and the median hospital stay was shorter for 18 patients who had early (within 15 days) recovered normal IGF-1 values (11 vs 18 days, p = 0.0589). According to the definition of Olthoff and colleagues[24], 11 (35.5%) patients experienced an EAD. Although a lower IGF-1 serum level was noticed in the EAD-group on postoperative days 15 (86.7±18.4 vs 111.9±15.0 ng/mL; p = 0.3073) and 30 (126.3±21.5 vs 164.0±20.7 ng/mL; p = 0.2580), this difference was not statistically significant at any of the considered time points. Six (19.4%) out of 31 patients experienced a biopsy-proven ACR after LT, at a median time of 17 days post-LT (range: 5–165). A mild histological ACR was diagnosed in the majority of cases (5/6, 83.3%), and treatment was based on steroid pulse therapy. Biliary stenosis was diagnosed in 5 (16.1%) patients after a median time from LT of 98 days (range: 11–297). Stenosis was treated conservatively by endoscopic retrograde cholangiopancreatography in all cases. By the end of one year, neither the presence of biliary stenosis (129.6±10.1 vs 103.3±33.2 ng/mL), nor the incidence of a biopsy-proven ACR (122.0±10.6 vs 142.6±23.4 ng/mL), significantly affected the IGF-1 plasma levels (p = 0.3791 and p = 0.3968, respectively).

**Fig 3:**
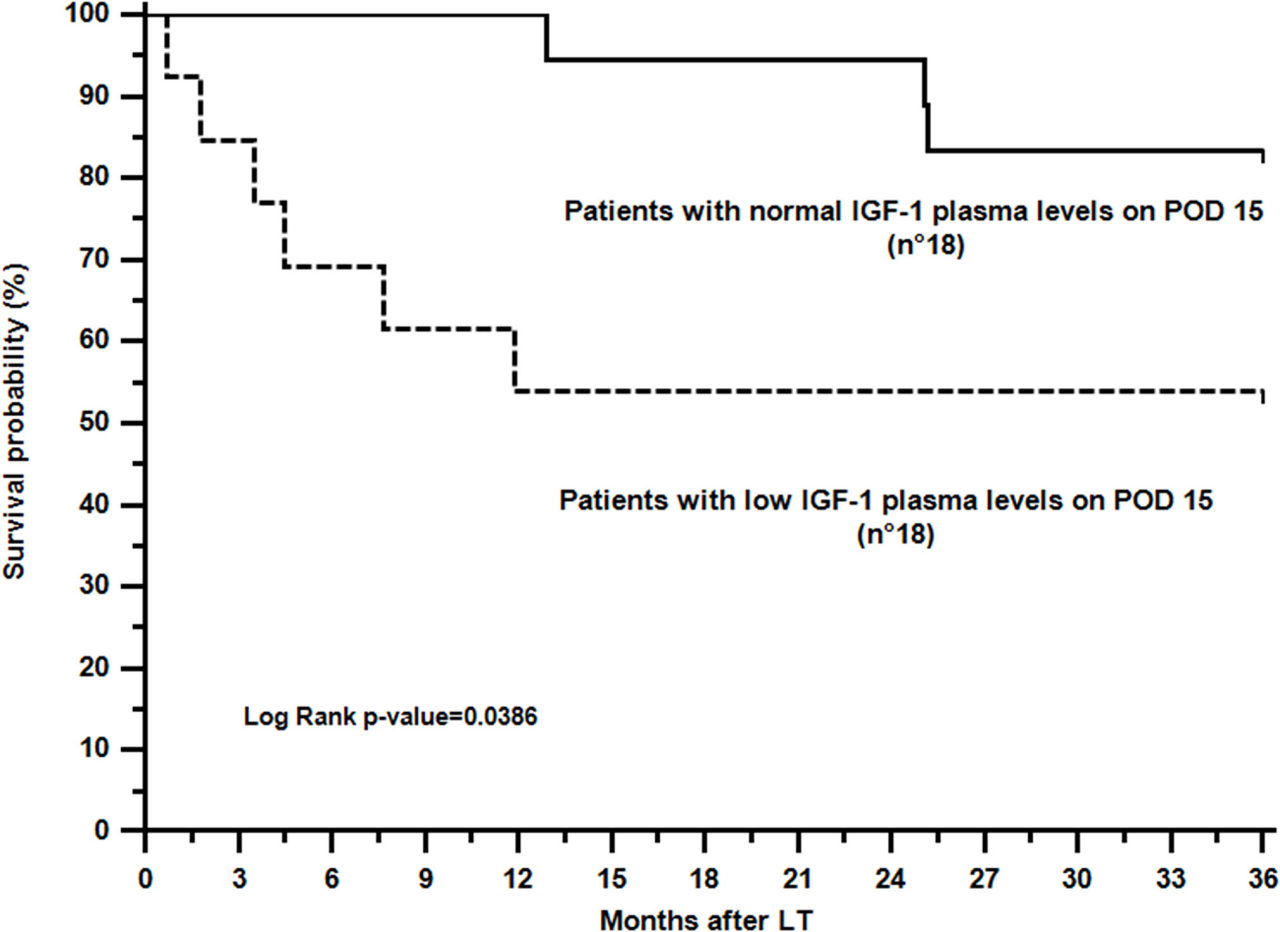
Actual 3-year survival analysis in patients showing normal (solid line) or low (dotted line) IGF-1 plasma levels on postoperative day 15. Survival differences were investigated by the Log Rank test. **Abbreviations:** IGF-1, insulin-like growth factor 1; LT, liver transplantation; POD, postoperative day.

At the end of follow-up, the survival rate of the entire cohort was 71%. The causes of death included graft dysfunction complicated by sepsis (3 cases), HCV recurrence (2 cases), late graft dysfunction (2 cases), thrombotic thrombocytopenic purpura associated with graft dysfunction (1 case) and late portal vein thrombosis due to an unspecified thrombophilic disorder (1 case). Interestingly, 7 out of 9 patients had abnormal IGF-1 levels at the scheduled visit before death occurred. Patients who promptly recovered normal IGF-1 plasma levels showed a statistically significant survival advantage of 3 years after LT (83.3% vs 53.8%, p = 0.0386; Fig 3). Considering the clinical variables shown in Table 3, early (within 15 days) IGF-1 normalization (Exp(b): 3.913; p = 0.0484) was the only independent prognostic factor associated with an increased 3-year actual survival rate.

**Table 3:** Univariate and multivariate analysis of risk factors affecting patient survival. IGF-1, insulin-like growth factor 1; LT, liver transplantation; ECD, extended criteria donor; DMELD, donor age x Model for End Stage Liver Disease; EAD, early allograft dysfunction; DRI, Donor Risk Index; HCV, hepatitis C virus; HCC, hepatocellular carcinoma; POD, postoperative day.

	Univariate analysis			Multivariate analysis	
Risk factors	3-years actual survival rate (%)	HR (95% C.I.)	Log Rank *p value*	Exp(b) (95% C.I.)	*p value*
Recipient Age ≥ 60 years	81.8 vs 65.0	0.5022 (0.129–1.954)	0.3806		
DMELD score ≥ 1300	57.1 vs 75.0	2.059 (0.396–10.698)	0.2956		
HCV-RNA positive before LT	58.3 vs 78.9	2.058 (0.535–7.916)	0.2715		
Presence of HCC	63.6 vs 75.0	1.410 (0.362–5.493)	0.6063		
Total Ischemia Time > 600 minutes	70.8 vs 71.4	0.948 (0.202–4.461)	0.9481		
Early Allograft Dysfunction	63.6 vs 75.0	1.735 (0.425–7.078)	0.4050		
ECD-score ≥ 2	80.0 vs 62.5	2.035 (0.551–7.518)	0.3039		
DRI-score ≥ 1.7	72.0 vs 66.7	0.790 (0.146–4.262)	0.7679		
Donor Age > 65 years	60.0 vs 90.9	5.587 (1.481–21.077)	0.0664	4.131 (0.492–34.652)	0.1912
Abnormal IGF-1 plasma levels on P.O.D. 15	53.8 vs 83.3	3.858 (0.973–15.300)	0.0386	3.913 (1.012–15.749)	0.0484

## Discussion

Changes in the GH/IGF-1 axis are well-documented in cirrhosis, and the peptides involved have been proposed as markers of hepatocellular dysfunction, malnutrition and survival in this category of patients[11, 25, 26]. Low IGF-1 circulating levels in parenchymal liver disease are mainly due to an impaired production associated with severe GH hepatic hormonal resistance[5, 9]. Our prospective study showed that LT recovers the normal GH/IGF-1 serum levels in most patients within the first year after surgery. Moreover, IGF-1 accurately reflected the graft’s functional reserve during the postoperative period, and for the first time, the prognostic role of hormonal recovery on patient survival was demonstrated in our series.

Within the context of liver cirrhosis, several studies have highlighted the strong correlation between basal and stimulated IGF-1 serum levels and the degree of liver failure, as expressed by the Child Pugh[11, 27] or MELD-score[9], serum albumin or INR. All patients included in our study had low IGF-1 plasma levels, and as recently observed by Castro and colleagues[16], a significant inverse correlation was observed comparing preoperative values and the uncorrected MELD score. Neither the etiology of liver disease nor the Child Pugh score correlated significantly with preoperative IGF-1 levels in our study.

The dramatic metabolic change represented by LT leads to a complete recovery of the GH/IGF-1 axis in adult and pediatric patients with end-stage liver disease[13–16, 28]. Based on our experience, we believe that the rise of IGF-1 plasma levels can be directly correlated with the graft’s functional recovery. To this regard, we noticed a significant correlation with common biochemical indicators of liver function, such as serum bilirubin and albumin, during the first months after LT. Even if some authors suggest that this hormonal increase could be an expression of hepatocyte damage or GH hyperstimulation[29], no significant correlation was found between IGF-1 and AST or GH at any of the time points considered in our study. In the current study, we did not investigate the modulatory activity of IGF-1 binding proteins (IGFBPs) after LT; however, two reports have demonstrated that the mean values of IGFBP-3, which is the most represented binding protein, increase to the high-normal range, which is consistent with IGF-1 restoration[16, 30]. This finding suggests that the total amount of IGF-1 in circulation determined by laboratory assays represents approximately a multiple of the unbound active IGF-1.

With regards to LT, most of the studies were focused on blood samples taken from months to years after surgery; therefore, no correlation between hormonal recovery and liver functional capacity could be investigated in the early postoperative period. For the first time, Bassanello et al. observed an immediate rise in IGF-1 levels after the graft’s reperfusion and the obtaining of the normal range within 1 month after surgery[17]. In that study, the 15 patients enrolled showed a complete hepatic recovery. As stated by the authors, the primary endpoint of that study was to explore IGF-1 dynamics in the early stages after LT, giving the same chance to every enrolled patient of achieving a GH/IGF-1 axis recovery. Consequently, strict inclusion criteria were applied, and cases with long ischemia time, elevated intraoperative blood loss and patients who received an ECD graft were excluded. For this reason, together with the short follow-up period, no certain conclusions could be drawn about the real capacity of IGF-1 to reflect the graft’s functional reserve and predict transplant outcome in the medium-term. In the era of the shortage of organs for transplantation, the use of “marginal” livers (retrieved from older, steatosic and unstable donors) is mandatory, and the reliability of a new marker of a graft’s quality should be tested over a heterogeneous population of donors while taking into account the main transplant variables related to the donor, recipient and postoperative course. In our series, the characteristics of liver donors (mean age of 67.2 years and more than half with 2 or more criteria of marginality) allowed us to recognize two different IGF-1 serum profiles between standard and ECD grafts during the first year after LT. The IGF-1 recovery curve was more sensitive in distinguishing the graft’s functional capacity from the third month up to 1 year after LT than the other LFTs that progressively returned to normal. Even if a successful LT can be often performed with ECD grafts to decrease overall waiting list mortality[31], it is to be expected that the liver functional reserve, and consequently graft survival in the long-term, could be different compared to when an optimal donor is employed. We found that IGF-1 is a reliable and clinically feasible method to monitor a graft’s function in this subcategory of patients.

In our experience, neither biliary complication nor acute cellular rejection significantly affected postoperative IGF-1 serum levels. As described, biopsy-proven ACR was classified as mild in 5 of the 6 patients, and treatment required exclusively steroid pulse therapy with no cases of steroid-resistant acute rejection. Similarly, biliary stenosis was promptly recognized in 5 of the 31 patients, and LFTs returned to normal within a few days after successful endoscopic treatment. The limited impact on the functionality of the graft of the aforementioned complications can explain the lack of significant modifications of IGF-1 levels in these patients.

The evaluation of liver functional reserve remains a critical issue before and after LT[18]. Standard liver biochemistry tests and clinical criteria may be controversial during this period, and new methods for evaluating graft status and patient prognosis early after transplantation are needed. In experimental and clinical studies, the amount of bile production has long been recognized as an important clinical parameter to predict EAD or PNF and to discriminate a graft’s quality between older (or ECD) and standard donors[32–34]. In our series, no significant differences in bile output emerged in patients receiving ECD or standard grafts. Recently, a study by Sutton and colleagues focused on normothermic perfused livers showed that a higher biliary secretion of bilirubin was found in grafts with high bile output. This finding suggests that bile quality, not only the volume, reflects the viability of the transplanted livers[35]. Thus, the determination of biliary IGF-1 concentration could be an attractive option to assess functional reserve and prognosis in future investigations. Other experimental methods, such as determination of liver fatty acid-binding protein (L-FABP)[36] and complement fragment 4d deposition[37], are suggested to be indicators of hepatocyte damage and predictors of initial poor function of the graft. Unfortunately, the majority of these techniques is time-consuming, hardly reproducible and cannot be routinely used in clinical practice. Similarly, even if several studies have shown that an impaired Indocyanine green clearance can predict early postoperative complications and transplant outcome, this method fails to provide relevant prognostic information on an individual patient basis because the uptake and excretion of Indocyanine green are influenced by many factors, such as cholestasis or hyperbilirubinemia in the perioperative phase[38–40]. The serum IGF-1 quantitative measurement, which is performed using chemiluminescent immunometric assay, is a standardized, reproducible and quickly available procedure. In addition, the presence of conjugated and unconjugated bilirubin has no effect on the test’s reliability, and the cost for each sample is approximately 9 euro.

Recently, Salso and colleagues explored the IGF-1 restoring process in 30 transplanted patients and demonstrated a positive correlation between cholesterol serum levels and the predictive value of IGF-1 for short-term survival (3 months) after LT[41]. The considerable follow-up of the patients included in our study (3 years) suggests that the recovery of IGF-1 within the second week after surgery identifies patients with favorable prognosis in the long-term period. In addition, multivariate analysis showed that IGF-1 early recovery was an independent predictor of outcome, independent of the quality of the graft (ECD-score or DRI), early postoperative graft function (according to the definition of Olthoff and colleagues) or other prognostic models such as DMELD.

In conclusion, this study is the first to demonstrate that a prompt recovery of IGF-1 serum levels is associated with long-term patient survival and shorter hospital stay in LT recipients. This marker was also confirmed to be a reliable index of hepatocellular functional reserve and was able to identify different patterns of recovery between standard and ECD liver grafts. Obviously, a larger number of patients is needed to confirm the clinical and prognostic significance of GH/IGF-1 axis recovery in the context of LT; however, on a purely speculative basis, our findings suggest that IGF-1 could represent a new donor-specific qualitative assay, identifying those grafts who are unable to withstand the injuries associated with procurement, cold preservation, and reperfusion and that result in a higher risk of graft failure.
